# RIPK2 is an unfavorable prognosis marker and a potential therapeutic target in human kidney renal clear cell carcinoma

**DOI:** 10.18632/aging.202808

**Published:** 2021-03-31

**Authors:** Diangeng Li, Liangyou Tang, Bo Liu, Shibo Xu, Meiling Jin, Wu Bo

**Affiliations:** 1 Urology Department, First Hospital of Shanxi Medical University, First Clinical Collage of Shanxi Medical University, Shanxi Medical University, Taiyuan 030001, China; 2 Department of Nephrology, Beijing-Chaoyang Hospital, Beijing 100020, China; 3 Office of Academic Research, Beijing-Chaoyang Hospital, Beijing 100020, China; 4 Department of Urology, West China Hospital, Sichuan University, Chengdu 610041, China; 5 Department of Medicine, Shenzhen University, Guangdong 518061, China; 6 Torch High Technology Industry Development Center, Ministry of Science and Technology, Beijing 100045, China; 7 Department of Nephrology, Chinese PLA General Hospital, Chinese PLA Institute of Nephrology, State Key Laboratory of Kidney Diseases, National Clinical Research Center for Kidney Diseases, Beijing 100853, China

**Keywords:** RIPK2, oncogene, kidney renal clear cell carcinoma, TCGA, immunity

## Abstract

*Receptor Interacting* Serine*/Threonine Kinase 2 (RIPK2)* is located on chromosome 8q21 and encodes a protein containing a C-terminal caspase activation and recruitment domain (CARD), which is a component of signaling complexes in both the innate and adaptive immune pathways. To estimate the value of RIPK2 in evaluating the prognosis and guiding the targeted therapy for patients with kidney renal clear cell carcinoma (KIRC), we analyzed total 526 KIRC samples from The Cancer Genome Atlas (TCGA) database. Our result showed that RIPK2 was upregulated in KIRC tumor samples compared with normal samples. Cox regression was performed to calculate the hazard ratio of RIPK2 expression as an unfavorable prognosis feature for overall survival. Moreover, RIPK2 expression was positively correlated to the high-risk clinical stage, and metastasis features. The upregulation of RIPK2 was strongly correlated with various immune signaling pathway dysregulations as well as immune phenotypes changes in KIRC patient’s cohort. In addition, inhibition of RIPK2 activity by either shRNA-mediated knockdown or inhibitor significantly reduced kidney cancer cell viability, trans-migration *in vitro*, and impaired tumor growth *in vivo*. In conclusion, elevated RIPK2 expression indicates a worse prognosis for KIRC patients and could serve as a potential prognostic biomarker and therapeutic target in kidney cancer.

## INTRODUCTION

The RIPK2 kinase transduces signaling downstream of the intracellular peptidoglycan sensors, nucleotide-binding, and oligomerization domain (NOD1) and NOD2, to promote a productive inflammatory response [1, 2]. The NOD1 and NOD2 are cytosolic Nod-like receptor (NLR) family proteins, activation of which will further activate NF-κB and MAP kinases, leading to the transcription of pro-inflammatory cytokines and the induction of autophagy [3]. Signaling by NOD2-RIPK2 has attracted wide scientific attention owing to its significant role in numerous diseases, making pharmacologic inhibition of *RIPK2* activity be a promising strategy [2, 4]. Accumulating studies have implicated that *RIPK2* predominantly expresses in the human breast, kidney, liver, and ovary tissues, and is upregulated in different types of tumors, including bladder, breast, and lung cancers [5–8]. As a remarkable association between the expression level of the RIPK2 gene and oncogenesis has been established, it has been shown as a potential target for cancer therapeutic intervention.

Kidney renal clear cell carcinoma (KIRC) is the eighth most common type of cancer and accounts for 70–80% of renal cell carcinoma, representing 4.2% of all new cancer cases, with about 73,820 new cases and 14,770 deaths estimated for 2019 in the United States [9]. Generally, there is no early clinical symptom revealed until the volume of the tumor is large enough. Thus, most KIRC patients are diagnosed in the medium or late stages, in which the mortality and recurrence rates are quite high. It is urgent to study the carcinogenesis and progression of KIRC and identify some biomarkers for the early diagnosis of KIRC [10, 11]. However, to date, limited is known about the oncogenesis and pathogenesis of KIRC, and few biomarkers for clinical use has been found. As far as we have known, the oncogenic and prognosis role of the *RIPK2* gene in KIRC has not been systematically analyzed, and additional investigations are merited.

As one of the largest cancer genomics databases, The Cancer Genome Atlas (TCGA) has profiled more than five hundred KIRC samples with genomic alterations, expression profiles, and clinical annotations data have been involved. The genomics, transcriptomics, and clinical data of the KIRC cohort (a total of 526 tumor samples) from the TCGA database were been explored to obtain a better understanding of the roles of the *RIPK2* gene in the carcinogenesis process of KIRC. We investigated the somatic mutations, copy number variation, expression profiling, immunological features of the tumor microenvironment, and clinical association significance of the *RIPK2* gene in this work. Our analysis demonstrated that RIPK2 upregulation correlated with signature genes of tumor immunity, clinical features of metastasis, and high histological grade, suggesting that RIPK is a potential oncogene and unfavorable clinical prognosis marker for KIRC.

## RESULTS

### Oncogenic role and prognosis value of RIPK2 upregulation

**Figure 1:**
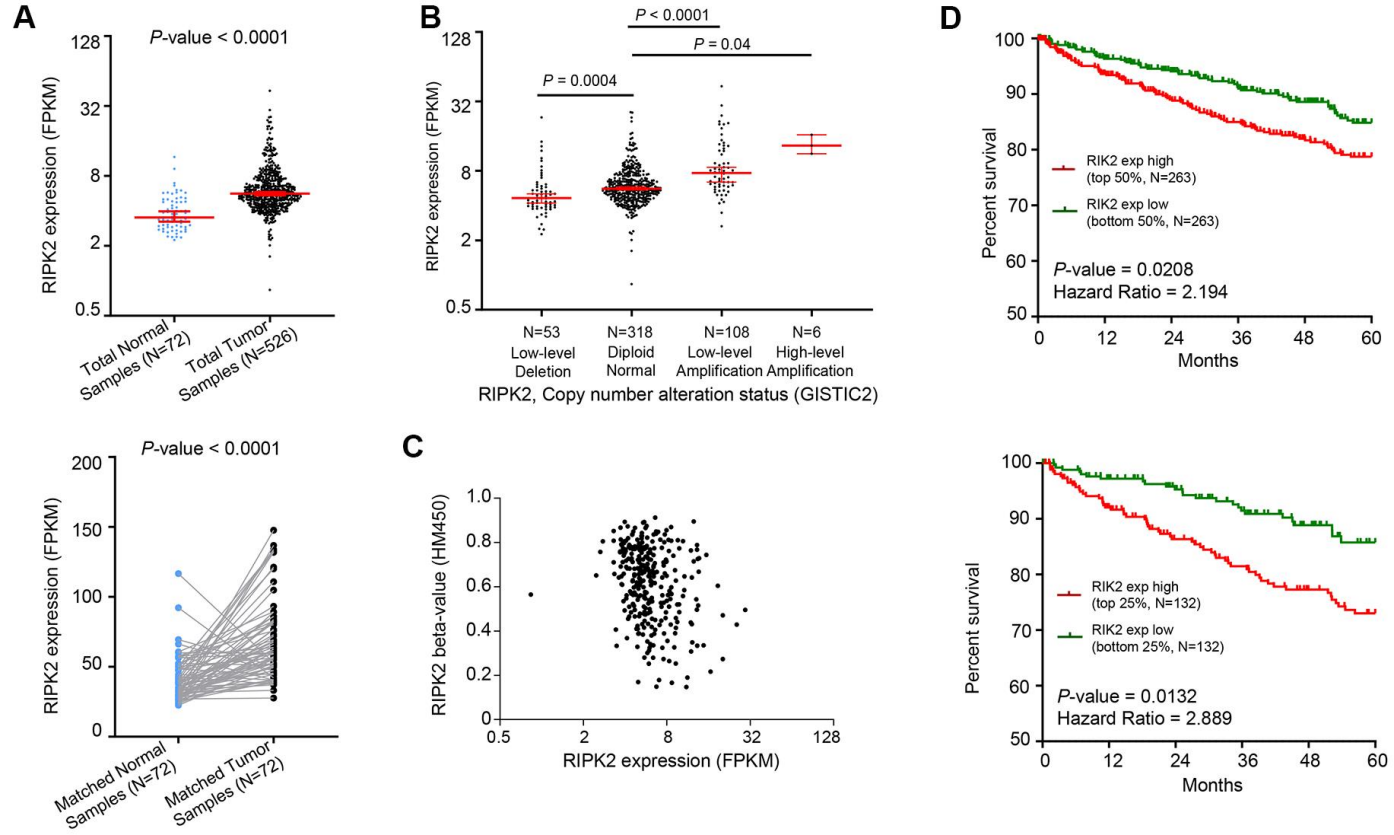
**Oncogenic role of *RIPK2* in human kidney cancer.** (**A**) Change of *RIPK2* mRNA expression between tumor samples and normal samples from TCGA KIRC studies. (**B**) Dot plot showing the positive correlation between *RIPK2* copy number values defined by GISTIC2 approach and mRNA expression values quantified by FPKM. (**C**) Dot plot showing the correlation between *RIPK2* methylation values defined by HM450 approach and mRNA expression values quantified by FPKM. (**D**) Kaplan–Meier survival curve comparing the high and low expression value of *RIPK2* (determined by the mean or quantile value) for the TCGA KIRC patient cohort. Statistical significance was determined by one-way ANOVA and the log-rank test.

First, the mRNA expression value of *RIPK2* was determined in KIRC samples based on RNA-sequencing data of the TCGA database. The results showed that the *RIPK2* gene was dramatically higher expressed in KIRC tumor samples (N = 526) compared with normal tissues (N=72) (Figure 1A, top, log-scale). The over-expression trend of *RIPK2* was also detected when compared with the matched tumor samples with adjacent normal samples (P < 0.0001) (Figure 1A, bottom, linear-scale). To further investigate whether the upregulation of *RIPK2* is due to the gene copy number amplification or epigenetics change (like promoter demethylated), we interrogated the chromosomal segment to determine the copy number alterations of KIRC samples. The KIRC patients could be stratified into four sub-groups based on the copy number values of gene *RIPK2* estimated by GISTIC2 algorithm (Low-level deletion sub-group, N = 64; Diploid sub-group, N = 389; Low-level amplification sub-group, N = 62; High-level amplification sub-group, N = 3) [12, 13]. The results revealed that about one-fifth of all KIRC samples harbored *RIPK2* copy number amplification (Figure 1B), and consistently, exhibited higher gene expression value than those harboring diploid RIPK2. In addition, we did not find any association between the methylation level at the gene region of *RIPK2* and its mRNA expression level (Figure 1C). Consequently, copy number gains of the gene *RIPK2* was likely the key machinery that contributes to the overexpression in KIRC patients. Furthermore, the upregulation of RIPK2 was also confirmed at both protein level, while the phosphorylation level of RIPK2 that representing it activated state was also significantly increased in KIRC samples (Supplementary Figure 2, 3).

Since the *RIPK2* gene was aberrantly upregulated in KIRC tumor samples, we next determined the prognostic value of its expression in KIRC patients. As shown in Figure 1D, the comparison of Kaplan–Meier curves (5-year overall survival) determined by the quartile (median, respectively) of *RIPK2* gene expression value revealed that higher expression sub-group was significantly correlated with inferior survival for KIRC patients. Taken together, a positive correlation between gene copy number amplification and over-expression of the gene *RIPK2* was observed in KIRC tumor samples, and its up-regulated expression could serve as an adverse prognostic marker for KIRC patients.

### RIPK2 upregulation is associated with the dysregulated immune pathway

**Figure 2:**
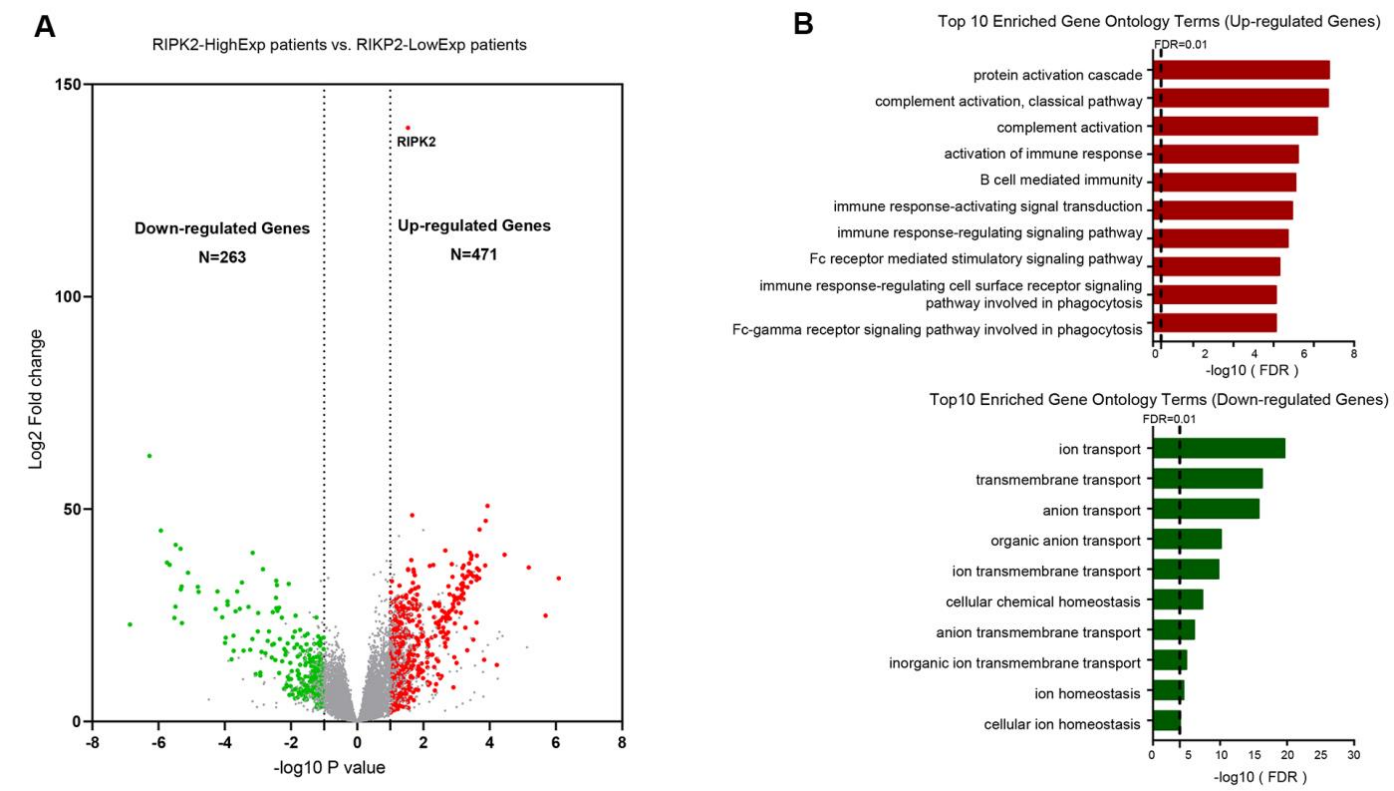
**Identification of differentially upregulated expressed genes.** (**A**) Volcano plot of mRNA expression changes between KIRC samples harboring *RIPK2* high- and low- expression value. The x-axis specifies the log2 fold-changes (FC) and the y-axis specifies the negative logarithm to the base 10 of the adjusted p-values. Gray vertical and horizontal dashed lines reflect the filtering criteria. Red and green dots represent genes expressed at significantly higher or lower levels, respectively. (**B**) Top 10 Gene ontology enrichment terms for up-regulated (top) and down-regulated (bottom) genes, respectively.

**Figure 3:**
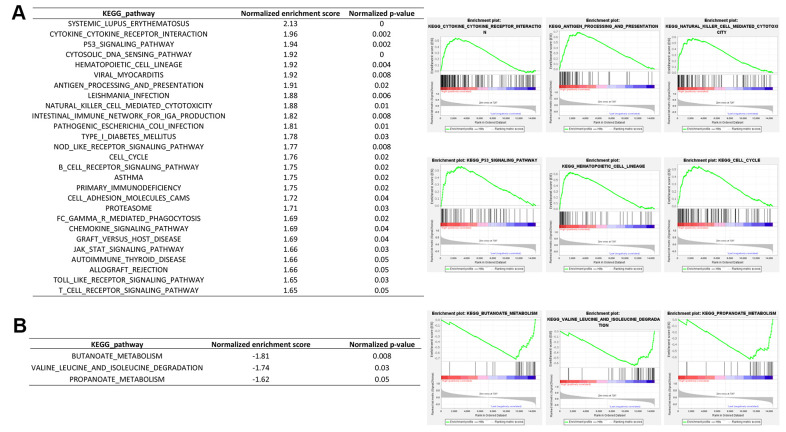
**Gene set enrichment analysis between *RIPK2* high- and low- expression samples.** GSEA comparing gene-expression signatures of TCGA KIRC tumors with the *RIPK2* high- and low-expression by using hallmark gene sets. GSEA positive result table (**A**) showing all the significant enrichment terms of the hallmark keg pathway gene sets from MSigDB, and GSEA negative result table (**B**) showing significant enrichment terms of the KEGG pathway gene sets from MSigDB.

We next performed the differential gene expression (DGE) analysis between *RIPK2* high-expression sub-group (N = 132) and *RIPK2* low-expression sub-group (N = 132) based on the KIRC cohort of the TCGA database (Figure 2). In total, we identified 471 significantly up-regulated genes and 263 down-regulated genes in *RIPK2* high expression samples when compared with low expression samples (Supplementary Table 1). And more interestingly, when we performed the gene ontology (GO) enrichment analysis by using these up-regulated genes as input, the results showed that nearly all of the enriched functional terms (9 of the top 10 enriched gene ontology terms) were immune-related (Figure 2B). Then, the gene set enrichment analysis (GSEA) by using the KEGG pathway gene sets from MSigDB database was also been employed (Figure 3A), which revealed that most of the immunologically related pathways (Cytokine-cytokine receptor interaction pathway, Antigen processing and presentation pathway, Natural killer cell mediated cytotoxicity, B cell receptor signaling pathway, T cell receptor signaling pathway) were positively enriched in samples harboring *RIPK2* high-expression compared with *RIPK2*-low expression samples (Figure 3A, Supplementary Figure 1). We also found a very limited number of significantly enriched metabolism-related gene sets (Butanoate metabolism pathway, Valine, leucine, and isoleucine degradation pathway, Propanoate metabolism pathway) were negatively enriched in *RIPK2* high-expression KIRC patients (Figure 3B). Consistently, most significantly KEGG pathway gene sets (the two of three) that enriched in sub-groups with *RIPK2* high-expression of KIRC patients were immune signaling related pathways (Figure 3B).

### Interaction pattern and immune phenotypes changes by RIPK2 upregulation in the kidney tumor microenvironment

**Figure 4:**
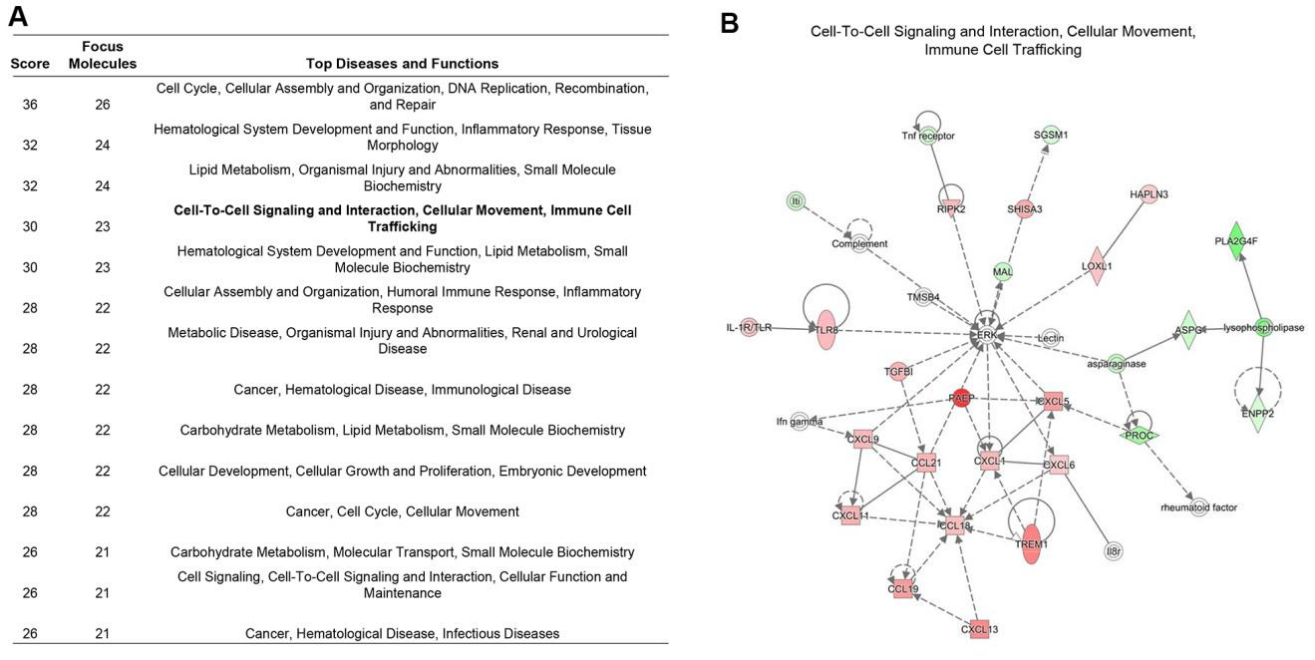
**Immune signaling interactions and network analysis.** (**A**) The table showed the significant signaling networks actively compared between the *RIPK2* high-, and low-expression samples by using Ingenuity Pathway Analysis (IPA) database. (**B**) The highest-ranked immunological signal pathway network revealed by IPA. Proteins indicated in red were up-regulated in RIPK2-high KIRC samples and the intensity of red means the foldchange. The shapes are indicative of the molecular class (i.e. protein family). Lines connecting the molecules indicate molecular relationships. In detail, dashed lines indicate indirect interactions, and solid lines indicate direct interactions. The style of the arrows indicates specific molecular relationships and the directionality of the interaction (**A** acts on **B**).

**Figure 5:**
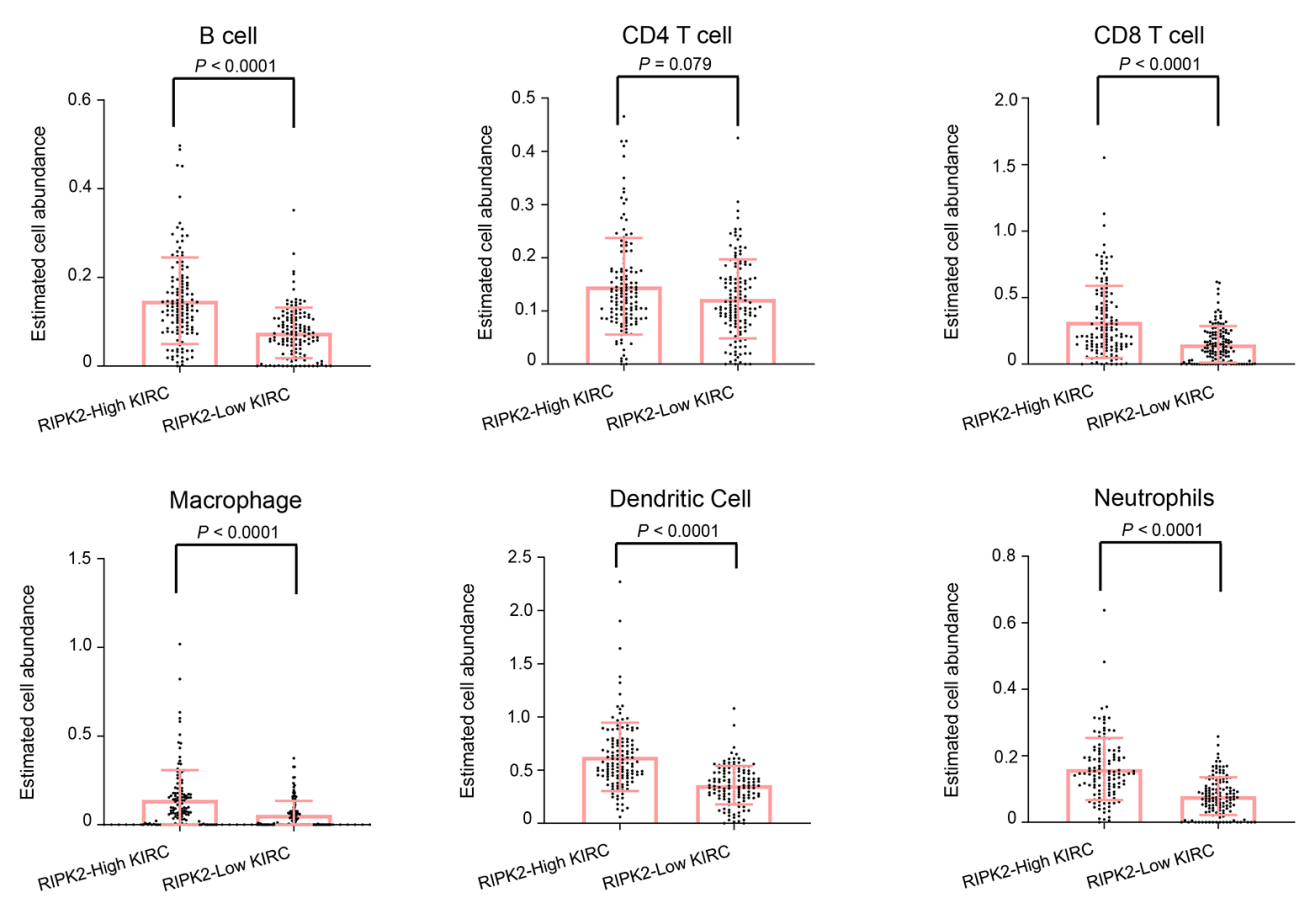
**The difference in estimated immune cell fraction.** Distribution plot of estimated immune cell fraction among between *RIPK2* high- and low- expression samples. Statistical significance was determined by the Wilcoxon rank-sum test.

The tumor microenvironment is a dynamic multi-scale molecular and cellular networks with high complexity that plays critical roles in tumor prevention, but also its initiation and progression. To determine the network-based interaction pattern change of the immunological activity, we next performed the pathways enrichment and signaling network analysis by using the Ingenuity Pathway Analysis (IPA) software. The identified significantly up-regulated genes in KIRC samples based on the above analysis were used as the input gene list. The results showed that among all the enriched networks, about half (7/14) are immune-related, which were consistent with differential expression analysis and GSEA pathway activity results (Figure 4A). The network representation of the high-ranked immunological signal network associated with “Cell-To-Cell Signaling and Interaction, Cellular Movement, Immune Cell Trafficking” was shown in Figure 4B. Collectively, these data demonstrated an obvious positive correlation between dysregulation of *RIPK2* gene expression and immunological changes in the tumor microenvironment. Considering KIRC is usually abundant in immune infiltrates consisting of lymphocytes, dendritic cells, macrophages, and others. Different functions are ascribed to the different subsets of immune cells. The role of the immune system changes in kidney cancer is not only observed through the inflammatory mediators but also at the cellular level, that is, via the changes of immune phenotypes and cellular interactions would lead to tumor microenvironment reorganizations. Furthermore, to investigate whether some specific subsets of leukocytes were affected by the *RIPK2* expression level, we estimated the abundance of each immune cell phenotype in the tumor microenvironment of KIRC by using the TIMER algorithm [14]. Among the major tumor-infiltrating immune effector cells that can be identified by TIMER, including T cell (CD4 T cells and CD8 T cells), B cells, macrophages, neutrophils, and dendritic cells. As shown in Figure 5A, most of the immune cell phenotypes had a higher infiltration level in RIPK2-overexpression samples compared with low expression KIRC samples, except for CD4 T cells.

### RIPK2 upregulation samples exhibit a selective increase of SETD2 and BAP1 genomic alterations

**Figure 6:**
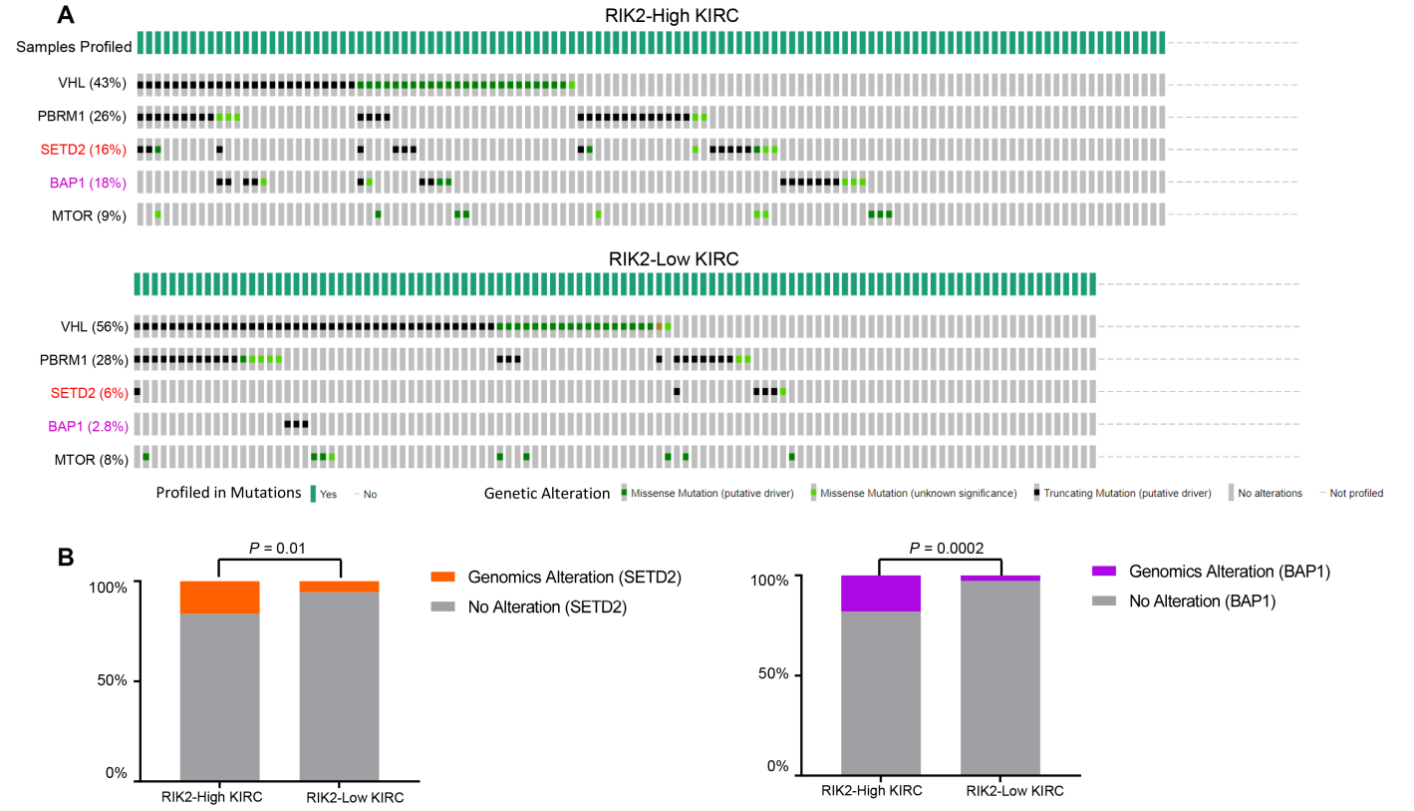
**SETD2 and BAP1 genomic alterations are selectively enriched in *RIPK2*-high expression samples.** (**A**) cBioPortal OncoPrint plot showing the distribution of VHL, PBRM1, CDKN2A, SETD2, BAP1, and MTOR genomic alterations rate in the TCGA KIRC dataset. (**B**) Bar graphs showing the percentage of TCGA KIRC samples with genomic alterations in SETD2 and BAP1 by different *RIPK2* expression groups.

There are many well-recognized hyper-mutated genes (VHL, PBRM1, SETD2, BAP1, and MTOR) in KIRC patients cohorts from the TCGA database. So we next evaluated the correlation of the *RIPK2* over-expression and the mutation profile of these genes to examine whether the tumor samples with *RIPK2* upregulation were enriched for some specific somatic alterations. The OncoPrint function (from cBioPortal for Cancer Genomics web-server toolkits, https://www.cbioportal.org/) was employed to investigate the somatic mutation rate of these top five altered genes in the TCGA KIRC cohort (Figure 6A). Interestingly, the results revealed that the alteration rate of SETD2 and BAP1 genes in the sub-group of KIRC patients with *RIPK2* high-expression is 3-fold higher than *RIPK2* low-expression sub-group (Figure 6B). Recent studies identified SETD2 functions as a tumor suppressor in kidney cancer, which was frequently inactivated and associated with the recurrence of clear cell renal cell carcinoma [15, 16]. For BAP1, it had been proven that BAP1 served as a tumor suppressor, and the loss of BAP1 could result in enhanced mesenchymal-epithelial transition in kidney tumor cells [17, 18]. However, the expression of both SETD2 and BAP1 was not affected by *RIPK2* knockdown (Supplementary Figure 4F, 5B, 5C).

### Associations between tumor clinical state and RIPK2 expression

**Figure 7:**
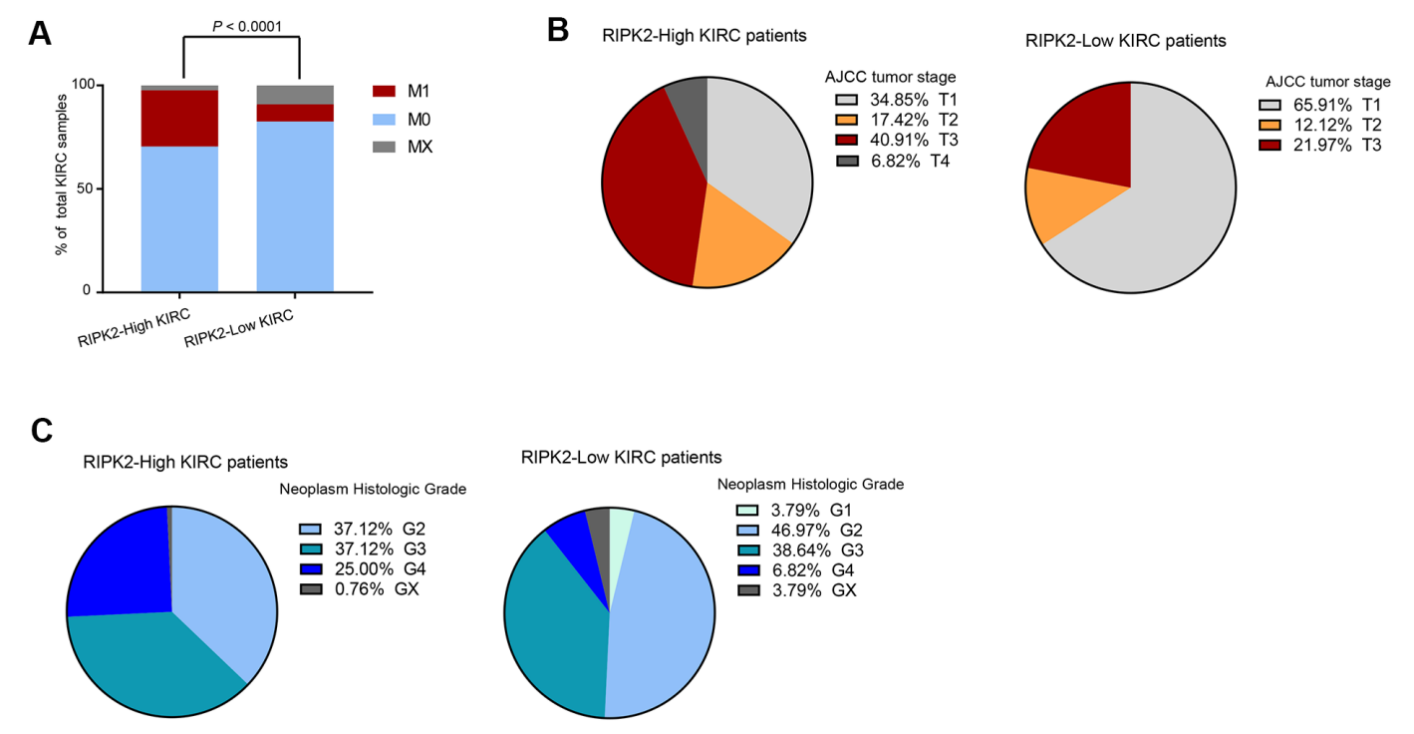
**The difference in clinical characteristics between *RIPK2* high- and low- expression samples.** (**A**) Distribution of M0 and MX samples among different *RIPK2* expression subtypes of KIRC from the TCGA patient cohort and functional modules activation analysis by IPA based on up-regulated genes in *RIPK2*-high samples. (**B**) Distribution of tumor stages among different *RIPK2* expression subtypes. (**C**) The distribution plot of the patient’s somatic mutation count (left) and the patient’s fraction of copy number altered (right) among KIRC patients between *RIPK2* high- and low- expression samples.

We next analyzed the distribution of clinical and pathological features among different *RIPK2* expression sub-groups. As expected, the tumor samples with metastasis status: M1 (spread to other parts of the body patients) were dramatically enriched in the sub-groups of KIRC patients harboring high-expression of the *RIPK2* gene (P < 0.0001, Figure 7A). Moreover, we also observed that there were dramatic differences in tumor stage (Figure 7B) and histologic grade (Figure 7C) distribution between high- and low-expression of *RIPK2* sub-groups. The tumors with *RIPK2* over-expression showed an increased risk of the high-grade stage when compared with *RIPK2* low-expression samples, and this was also confirmed at protein levels of both total protein and phosphorylation state (Supplementary Figure 3).

### Inhibition of RIPK2 activity suppressed cell proliferation *in vitro* and *in vivo*

**Figure 8:**
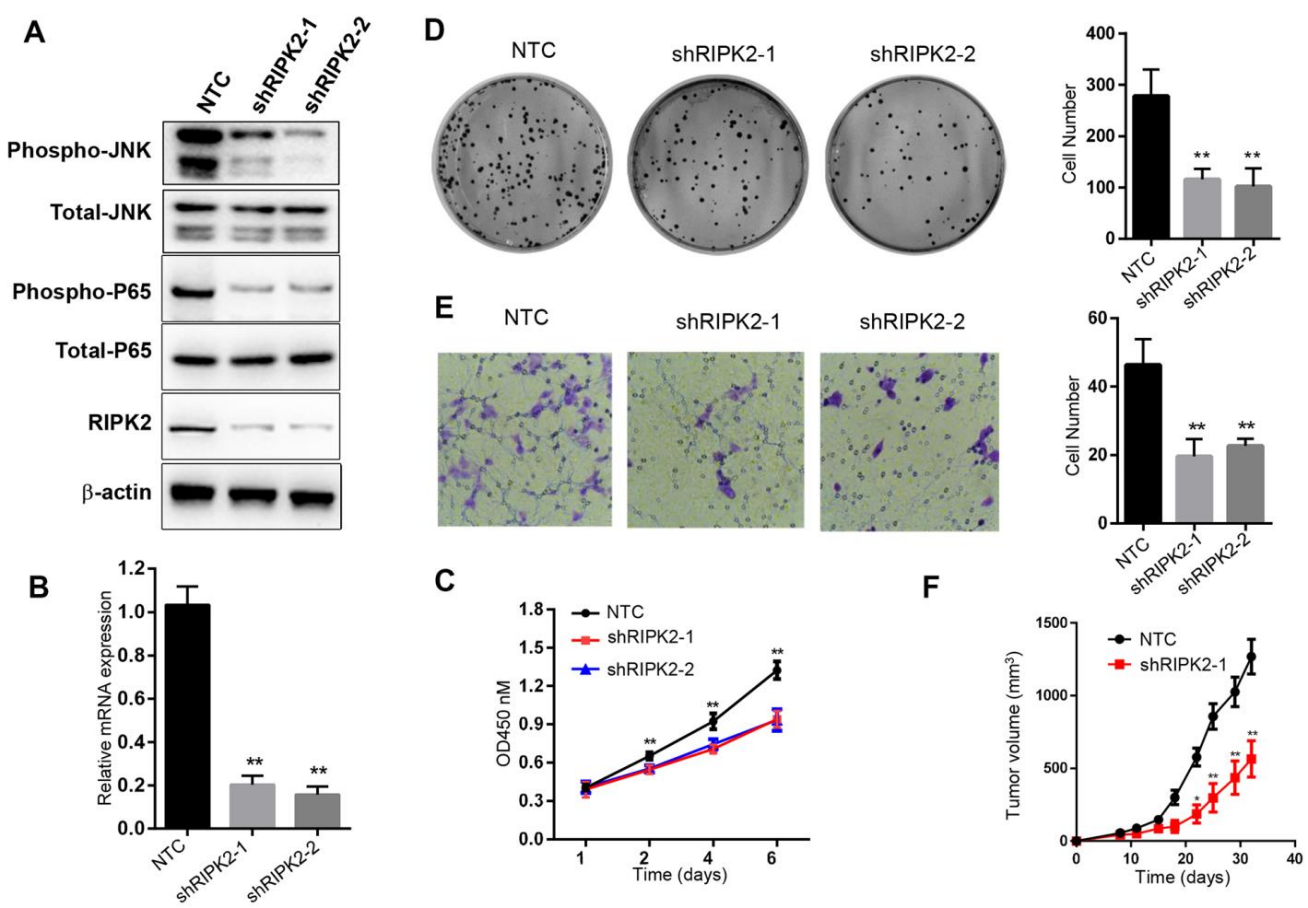
**Knockdown of RIPK2 inhibit the 786-O cell proliferation, migration, colony formation and *in vivo* growth.** (**A**) Western blot analysis of the 786-O cells stably transfected with control shRNA (NTC), or that targeting RIPK2 coding region (shRIPK2-1 and shRIPK2-2). (**B**) Quantitative PCR analysis of the *RIPK2* mRNA level. Data are means ± SD (n=3), **p<0.01; (**C**) Cell proliferation results expressed in means ± SD (n=5), **p<0.01. (**D**) Colony formation assay photos (left) and quantitative results (right), Data are means ± SD (n=3), **p<0.01. (**E**) Migration result of 786-O cells, migrated cells were calculated and expressed with means ± SD (n=5), **p<0.01. (**F**) *In vivo* xenograft model of 786-O, the tumor volume was determined twice a week. Tumor volume was expressed in means ± SEM (n=5), **p<0.01.

To investigate the relationship between RIPK2 expression and tumorigenic capacity, we firstly knocked down RIPK2 expression by shRNA strategy. Realtime PCR and Western blot results showed that the expression level of RIPK2 was reduced to about 20% by two different shRNA (Figure 8A, 8B). Cell count kit 8 assay and colony formation assay were performed to determine the ability of cell proliferation after RIPK2 knockdown. We found that decreased RIPK2 expression significantly delayed cell proliferation, and colony formation capacity (Figure 8C, 8D). Moreover, cell migration ability was also attenuated after reducing RIPK2 expression (Figure 8E). Mechanistically, RIPK2 knockdown greatly decreased the phosphorylation level of NF-κB and c-Jun N-terminal kinase (JNK), while no change was observed in the total protein levels of NF-κB and JNK. In a complemental experiment, GSK2983559 (3 μM), a selective inhibitor of RIPK2, was used to suppress RIPK2 activity. Expectedly, inhibition of RIPK2 by 3 μM GSK2983559 completely reproduced the effect of RIPK2 knockdown in cell proliferation and migration (Supplementary Figure 4B–4D). Furthermore, the combination of shRNA targeting RIPK2 and GSK2983559 showed no additive inhibitory effect on cell proliferation, thus excluding the possibility for off-target. To evaluate whether RIPK2 can be used as a potential therapeutic target in KIRC, we established the xenograft model by injection of RIPK2-knockdown or control cells into nude mice and monitored the tumor size over time. We observed that, the tumor size of nude mice in the RIPK2-knockdown group was significantly smaller, suggesting that inhibition of RIPK2 activity effectively suppress tumor growth *in vivo* (Figure 8F). Consistently, administration of GSK2983559 also suppressed the *in vivo* tumor growth (Supplementary Figure 4E).

## DISCUSSION

Previous studies have reported that the gene *RIPK2* played important roles in both inflammatory activity and tumor invasion and metastasis processes [4, 19]. Serine/threonine/tyrosine kinase contributes to the modulation of innate and adaptive immune responses and is involved in the tyrosine phosphorylation of the guanine exchange factor ARHGEF2 leading to NFκB activation by NOD2 [1, 20]. To our knowledge, this is the first demonstration of *RIPK2* upregulation during carcinogenesis in human kidney cancers. Our results highlighted the oncogenic role of *RIPK2* over-expression in KIRC and suggested that the copy number amplification would be one of the potential genetic drivers for its upregulation. Furthermore, our data revealed that *RIPK2* could serve as a potential unfavorable prognosis biomarker for KIRC patients. In the past few years, even though significant progress in biomedical imaging technology and application has greatly improved the diagnosis of renal cell carcinomas, most of these malignancies are still detected at an advanced stage due to their asymptomatic nature. Consequently, more oncogenes, particularly potential biomarkers for diagnosis and/or prognosis of KIRC are urgently required in this field.

*RIPK2* has been associated with activation of the NF-κB, JNK, extracellular signal-regulated kinase (ERK), and mitogen-activated protein kinase (p-38) pathways. *RIPK2* also mediates pro-inflammatory signaling from the bacterial sensors NOD1 and NOD2 and is an emerging therapeutic target in autoimmune and inflammatory diseases [19, 21, 22]. Interestingly, a strong correlation between *RIPK2* over-expression and dysregulated tumor immune infiltration level was found in this work. *RIPK2* promoted various immunological signaling pathways and immune phenotypes (including CD8 T cell, Dendritic cell, B cells, Neutrophils, and Macrophages) in the tumor microenvironment. The tumor microenvironment with chemotactic characteristics would recruit infiltrating immune cells including lymphocytes (such as T cell, B cell, and NK cell) and myeloid cell (such as monocyte, macrophage, and neutrophil). Usually, these infiltrating immune cells serve as a protective antitumoral role in the tumor microenvironment, but they could also contribute to cancer progression under particular circumstances. As exemplified by the tumor-associated macrophages (TAM), they could release angiogenic factors and fibroblast growth factors, which result in tumorigenesis and promote the process of tumor growth, invasion, and apoptotic resistance [7, 13, 23]. Thus, high infiltration of immune cells in kidney tumor samples may support tumors as that tumor cells, in turn, modulate the microenvironment within which they reside. Given that our work showed the potential linking between *RIPK2* dysregulated expression and altered immunological activities in the tumor microenvironment of KIRC, we believe that the increase of *RIPK2* transcripts would cause NF-κB signaling activation and subsequently contributing to KIRC pathogenesis and aggressiveness. The mechanism is still unclear what causes these links but the results presented lead us to suggest the possible use of *RIPK2* as a therapeutic marker, which will shed light on the future development of anti-cancer therapies for KIRC.

In this work, it is a very interesting and promising finding that our results showed the KIRC samples harboring *RIPK2* over-expression enriched in the somatics mutants of gene *SETD2* and *BAP1*, these results provided potential interaction between *RIPK2* and these two well-known oncogenes of kidney cancer [24, 25]. As far as we have known, the gene *RIPK2* is not the target gene of *SETD2* and *BAP1*, and there is no published work that has leaked the possible correlation between them, so it will be an open question for the researcher in this filed. Besides, our data revealed that the copy number amplification probably provides a genetic fix that facilitates *RIPK2* overexpression in the KIRC cohort of this study, however, we believe that the gene amplification that we mentioned in this work would not the only mechanism to up-regulate the mRNA expression of the gene *RIPK2* in the cancer cells of KIRC. More exploratory and integrative cancer genomics work based on KIRC (such as epigenomics analysis) datasets will be needed in future work.

## CONCLUSIONS

Our findings identified *RIPK2* as a potential oncogenic gene for KIRC and revealed its involvement in dysregulated immunological activities in the tumor microenvironment of KIRC. We also proved that *RIPK2* could serve as a novel prognosis marker and therapeutic target in KIRC. In future clinical use, the anti-RIPK2 agents as a potential therapy for KIRC patients may open new avenues for the development of antineoplastic drugs.

## MATERIALS AND METHODS

### Plasmids and reagents

shRNA plasmids. RIPK2 shRNA sequence was cloned into SHC201 vector (Sigma-Aldrich) and the plasmids were verified by DNA sequencing. The shRNA primer sequences are as follows: shRIPK2-1: 5′- GGA CAT CGA CCT GTT ATT AAT -3′; sh RIPK2-2: 5′- CAC CAA TCC TTT GCA GAT AAT-3. Antibodies for RIPK2 (ab75257), JNK1+JNK2+JNK3 (ab179461) and JNK1 + JNK2 + JNK3 (phospho Thr183+Thr183+Thr221) (ab124956) were purchased Abcam, and the antibody for β-actin (A5441) was obtained from Sigma-Aldrich; NF-κB p65 (# 8242), Phospho-NF-κB p65 (Ser536) (# 3033) were purchased from Cell Signaling Technology, Inc. GSK2983559 (# S8927) was from Selleck.

### Cells and cell culture

Human KIRC cell line 786-O cells (CRL-1932™) and HEK293T cells (CRL-11268™) were obtained from ATCC. 786-O cells were cultured in RPMI-1640 medium (Gibco, 31870082). HEK293T cells were cultured in DMEM medium (Gibco, C11965500BT). The complete culture media were composed of 10% heat-inactivated fetal bovine serum (FBS) (Gibco, 10099-141), 1% antibiotics mixture of penicillin and streptomycin (Gibco, 15070063) and 2 mM glutamine (Gibco, 15140-122). Cells were maintained in a wet incubator at 37° C with 5% CO_2_. Mycoplasma contamination was tested by PCR.

### Lentivirus package

The recombinant SHC201 vectors were co-transfected in HEK293T cells with PMD2.G (Addgene, #12259) and psPAX2 (Addgene, #12260) using Lipofectamine 3000 (Life Technologies) for lentivirus packaging. Then the virus particles were harvested and used to infect 786-O cells. And the infected cells were selected in media supplemented with 5 μg/ml of puromycin for 3 days. SHC201 empty vectors were used as controls.

### Cell count kit 8 assay

Cell Counting Kit 8 (Cat. No C0038) was purchased from Beyotimes and used to evaluate cell proliferation. Briefly, 2000 cells were seeded into 96 well plates and cultured with 100 μl complete culture medium. Cell activity was analyzed by adding 10μl CCK8 reagent to each well at day 1, 2, 4 and 6. Multiskan™ FC (Thermo) was used to detect the value of OD450 at each well of the plates after incubation for 1 hour. Experiments were performed in triplicate.

### Colony formation assay

786-O cells were cultured in six-diameter dish (500 cells / well) with 4ml complete medium. After 2 weeks incubation, the cells were washed with cold phosphate buffer saline (PBS) and then fixed with methanol for 30min, followed by incubation with crystal violet dye for another 30 min, colonies were imaged and counted for analysis.

### Transwell assay

24-well chemotaxis chambers (Corning, #3422) were used to analyze migration potential. 10,000 cells were resuspended in 100 μl serum-free medium and then were added into the top chambers. the bottom chamber was filled with 600ul complete medium. 24 hour later, the membranes were firstly fixed with methanol for about 30 minute at 23° C, ant then were stained with crystal violet for 30 minutes. The migrated cells were imaged and counted in six independent fields per group using a microscope.

### qPCR

The total RNA was extracted from 786-O using Trizol (InvivoGen, 15596018) based method according to the manufacturer’s instructions. The first strand of cDNA was synthesized from 1 μg of total RNA with random primers and MMLV reverse transcriptase (Vazyme, R021). The polymerase chain reaction were performed with the CFX-96 Real-Time PCR System (Bio-Rad). The Glyceraldehyde-3-phosphate dehydrogenase (GAPDH) was chosen as an internal control for normalization, and the ΔΔCt method was used to analyze the relative expression of the analyzed genes. Each sample was measured in duplicates. The primer sequences are as follows: GAPDH: sense, 5′- GAA GGT GAA GGT CGG AGT C-3′; antisense, 5′-GAA GAT GGT GAT GGG ATT TC-3′; RIPK2: sense, 5′- CAG AAG CCT GCC TTA ACC -3′; antisense, 5′- CTT GGA TGT CAG TAG TGT CTA -3′; SETD2: sense, 5′- AGA CAG CAG AAG CAG ACA -3′; antisense, 5′- GCA CTG GAC GAT GAA CTG -3′; BAP1: sense, 5′- AAG GAG GAG GTA GAG AAG AG -3′; antisense, 5′- TGA GCC AGC ATG GAG ATA -3′.

### Western blot

The 786-O cells were collected and resuspended in lysis buffer (20mM Tris, pH7.5, 150mM NaCl, 5mM EDTA, 0.5% NP-40, 10% glycerol, protease inhibitor cocktail (Roche) at 4° C for 30 minutes, followed by centrifugation (12,000 × g for 15 minutes, at 4° C). The protein concentration was determined by Bicinchoninic acid (BCA) assay. Proteins were separated by SDS–PAGE and immunoblotted with anti-RIPK2 or anti-β-actin antibody.

### Animal experiment

All procedures of the animal experiment were approved by the Animal Experiment Ethics Committee of General Hospital of the Chinese People's Liberation Army. Female athymic BALB/c nude mice (aged 5-6 weeks) were purchased from the Vital River Laboratory Animal Technology Co. Ltd. To establish a xenograft model, nude mice were randomized into two groups and 5 × 10^6^ 786-O cells were respectively injected subcutaneously into the right flanks of mice of each group. An electronic caliper was used to measure the length and width of each tumor, and tumor volume was estimated by applying the following equation: volume = length × width^2^/2. For *the in vivo* pharmacodynamic model, GSK2983559 was administrated at 50 mg/kg b.i.d doses.

### Datasets

The genomics, transcriptomics, and clinical information data of the KIRC cohort were accessed from the TCGA database (Figure 1). Through the TCGA data portal (https://portal.gdc.cancer.gov/, March 2020), the masked copy number segment profiles and mRNA expression quantification profiles (HTSeq–FPKM) of all the KIRC tumor samples were obtained. The GISTIC2 algorithm was used to determine the gain or loss of copy number for each gene by transforming the DNA segment data with a noise threshold [26]. The detailed clinical information and annotated mutation files of KIRC tumor samples were accessed from cBioPortal for Cancer Genomics web-server (http://www.cbioportal.org/index.do, February 2020).

### Gene-set enrichment analysis

The gene-set enrichment analysis (GSEA) in this work was performed by using the GSEA software (version 3.0) [27]. The gene signatures that summarize the biological functions and pathway processes were accessed from the Broad Molecular Signatures Database (MSigDB v6.0, https://www.gsea-msigdb.org/gsea/msigdb/).

Then the hallmark gene-sets (N=50) and KEGG pathway gene-sets (N=186) were used as input for GSEA software. The signal-to-noise metric (with default parameters) was employed to rank all genes, and the 1,000 permutations were performed for the estimation of statistical significance.

### Survival analysis

To describe the effect of categorical or quantitative variables on overall survival time (5-year), the Kaplan-Meier curve with quantile level (the top 25% and the bottom 25%) as the cut-off value was performed, and the log-rank test was used to estimate the significance among multiple curves. The Cox proportional hazard regression model (5-year overall survival) was employed to perform the univariate survival analysis for the prognostic significance of each feature.

### Biostatistical analysis

For comparisons, the Mann-Whitney test (between two groups) and the one-way analysis of variance (ANOVA) test (among multiple groups) were used for statistical significance estimated. For the correlation analysis between two continuous variables, the correlation coefficient (Spearman) was calculated to estimate the significance of the association. For the enrichment analysis, Fisher's exact test was employed for significance estimation. For the differential expression analysis, the DESeq2 algorithm was used to perform between *RIPK2*-high and *RIPK2*-low KIRC samples [28] (False Discovery Rate (FDR) <0.05), log_2_ratio of expression mean value>1, and the difference of the mean value of normalized counts for each gene >500) to determine the significant difference. All the statistical calculations were performed using R software (https://www.r-project.org/) and the graphs display were performed by using GraphPad PRISM software (version 8.3, GraphPad Software, Inc.).

### Ingenuity pathway analysis

The Ingenuity Pathway Analysis (IPA) software was used for pathway enrichment and signaling network analysis in this work. The significantly up-regulated gene expression profile (with fold-change and P-value) based on DEG analysis was employed for the IPA software as the input gene list. Then the two functional models (1, Canonical Pathways, and 2, Signaling Network Analysis) of IPA software were run with default parameters.

### Availability of data or materials

The datasets used for the current study are available upon reasonable request from the corresponding author.

## Supplementary Material

Supplementary Figures

Supplementary Table 1
