# Supplementary material for: RIPK2 is an unfavorable prognosis marker and a potential therapeutic target in human kidney renal clear cell carcinoma
Source: Aging (Albany NY). 2021 Mar 31;13(7):10450–67. doi: 10.18632/aging.202808 (PMC8064209; doi:10.18632/aging.202808)
Supplement: Supplementary Figures [file aging-13-202808-s001.pdf]

SUPPLEMENTARY FIGURES

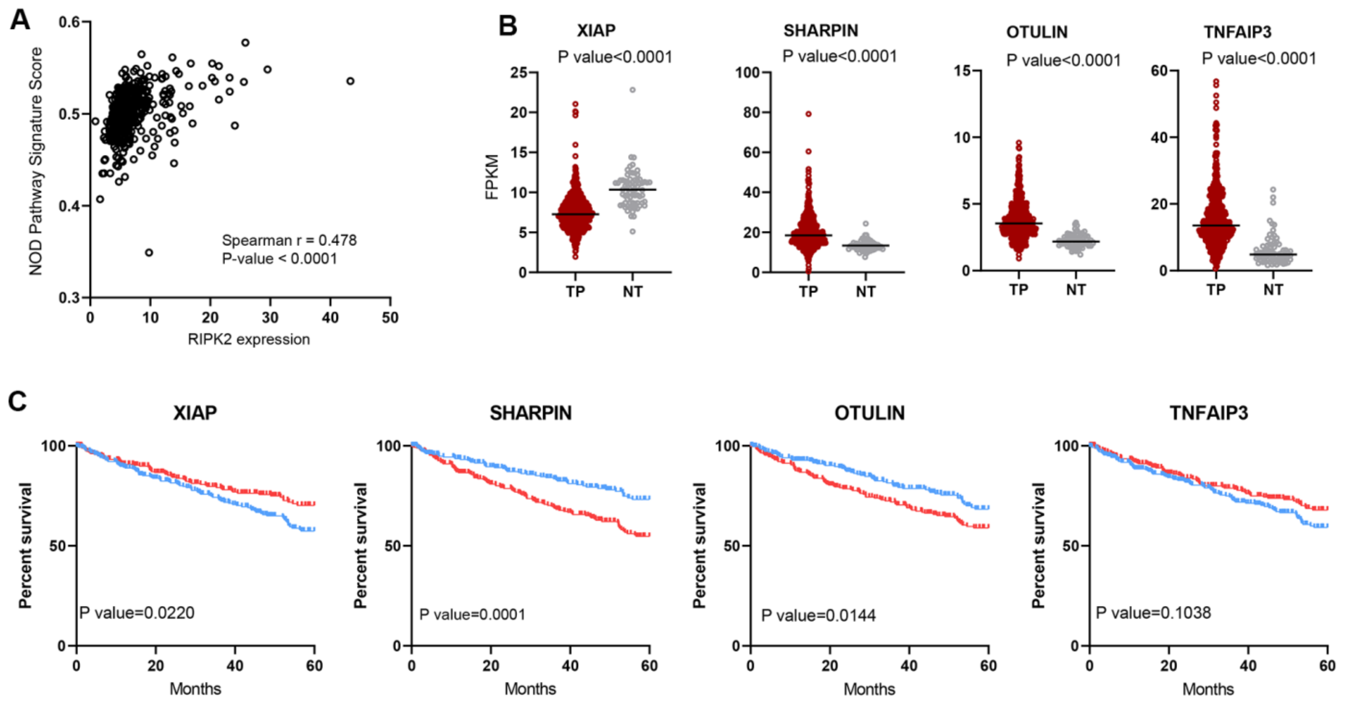

Supplementary Figure 1. Role of RIPK2 related pathway in KIRC. (**A**) The association plot of RIPK2 expression and NOD pathway activity. (**B**) The violin plot of the gene expression value of XIAP, SHARPIN, OTULIN, and TNFAIP3 in TCGA KIRC dataset. (**C**) The survival curve of XIAP, SHARPIN, OTULIN, and TNFAIP3 in TCGA KIRC dataset.

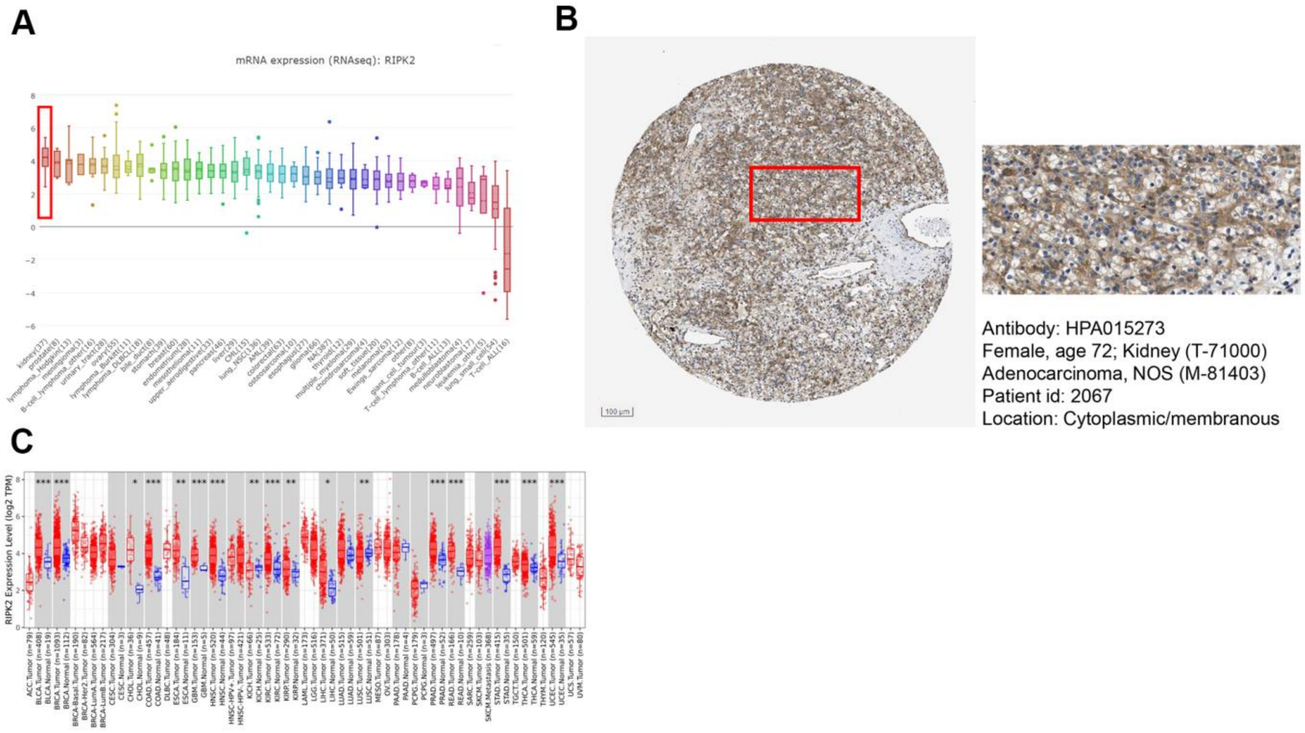

Supplementary Figure 2. Expression of RIPK2 in KIRC and other cancer types. (**A**) Expression profile of RIPK2 in cancer cell lines (Broad Institute Cancer Cell Line Encyclopedia Database). (**B**) Immunostaining of RIPK2 in renal cancer tissue, antibody: HPA015273, Patient ID:2067; Female, age: 72. (**C**) Expression of RIPK2 in different cancer types; *: p-value < 0.05; **: p-value <0.01; ***: p-value <0.001 (www.timer.cistrome.org).

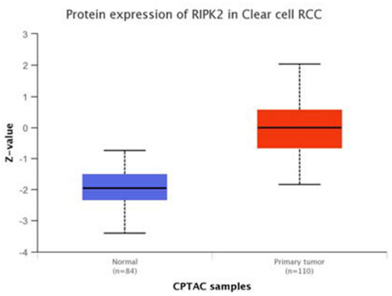

Comparison	Statistical significance
Normal-vs-Primary	5.82E-39

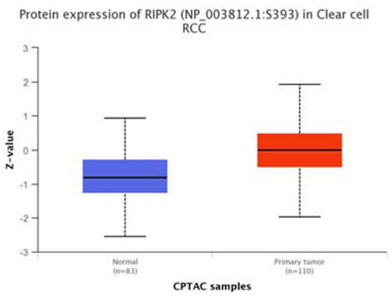

Comparison	Statistical significance
Normal-vs-Primary	1.05E-16

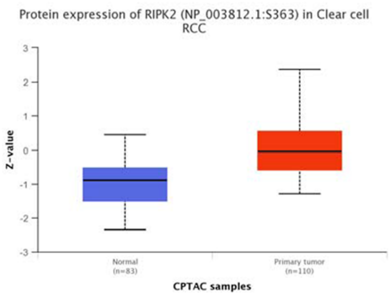

Comparison	Statistical significance
Normal-vs-Primary	4.49E-08

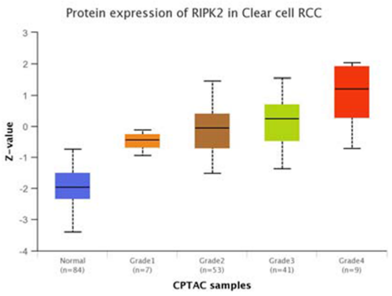

Comparison	Statistical significance
Normal-vs-Stage1	2.44E-23
Normal-vs-Stage2	1.79E-11
Normal-vs-Stage3	1.65E-10
Normal-vs-Stage4	1.29E-06

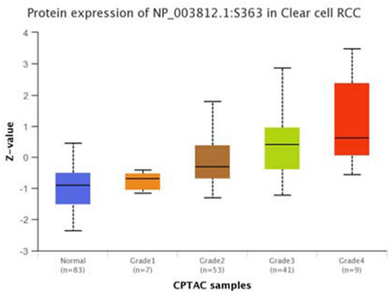

Comparison	Statistical significance
Normal-vs-Stage1	1.31E-01
Normal-vs-Stage2	9.19E-09
Normal-vs-Stage3	8.35E-11
Normal-vs-Stage4	2.14E-03

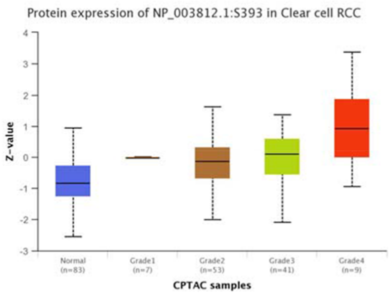

Comparison	Statistical significance
Normal-vs-Stage1	7.99E-05
Normal-vs-Stage2	1.06E-03
Normal-vs-Stage3	1.71E-04
Normal-vs-Stage4	9.53E-02

Supplementary Figure 3. Expression of RIPK2 and its phosphorylation in KIRC. Z-values represent standard deviations from the median across samples for the given cancer type. Log2 Spectral count ratio values from CPTAC were first normalized within each sample profile, then normalized across samples.

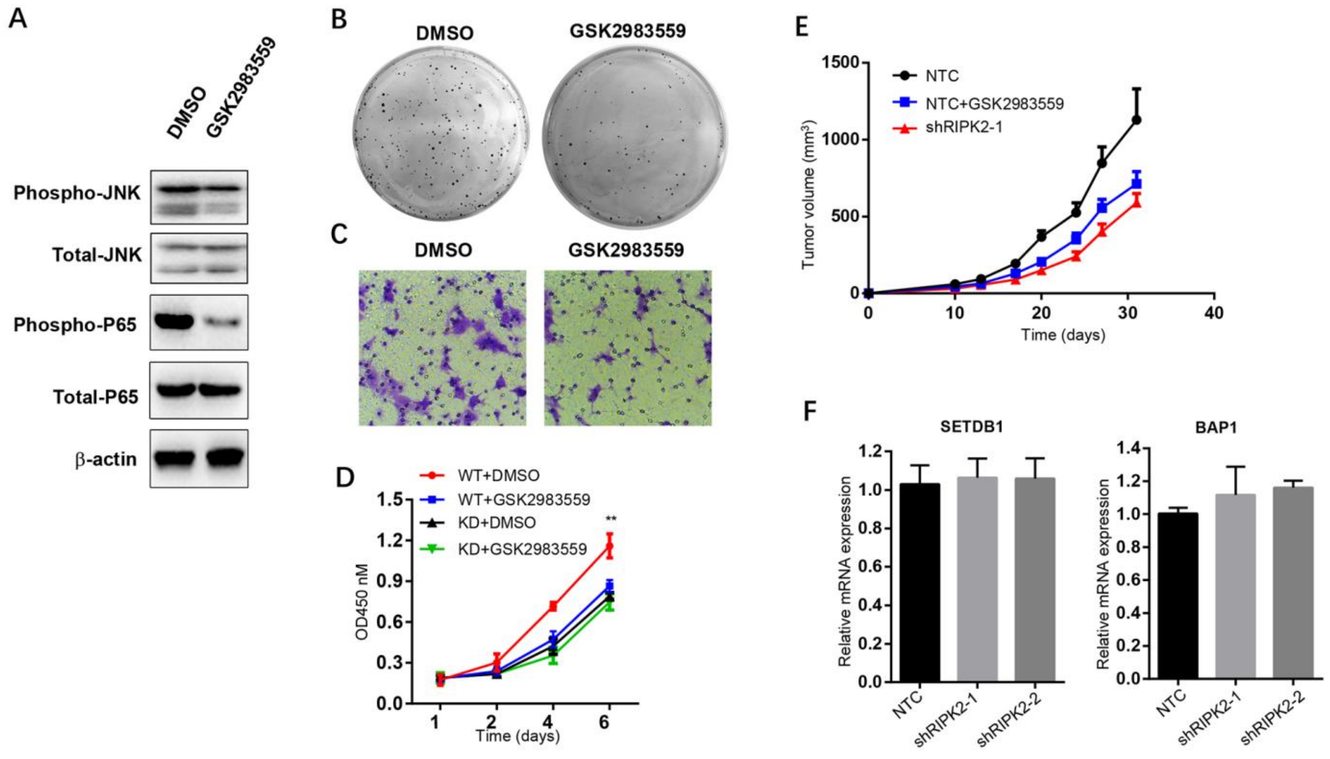

Supplementary Figure 4. Effect of RIPK2 inhibition on signaling pathway and *in vivo* tumor growth. (**A**) Western blot analysis of JNK and NFκB expression in 786-O cells upon treatment with GSK2983559. (**B**–**D**) GSK2983559 (3 uM) mediated RIPK2 inhibition leads to suppressed cell growth determined by colony formation assay (**B**), CCK-8 assay (**D**) and cell migration (**C**). (**E**) *In vivo* efficacy of GSK2983559 on 786-O xenograft model. (**F**) Quantitative PCR analysis of the SETD2 and BAP1 mRNA level. Data are expressed as means ± SD (n=3).

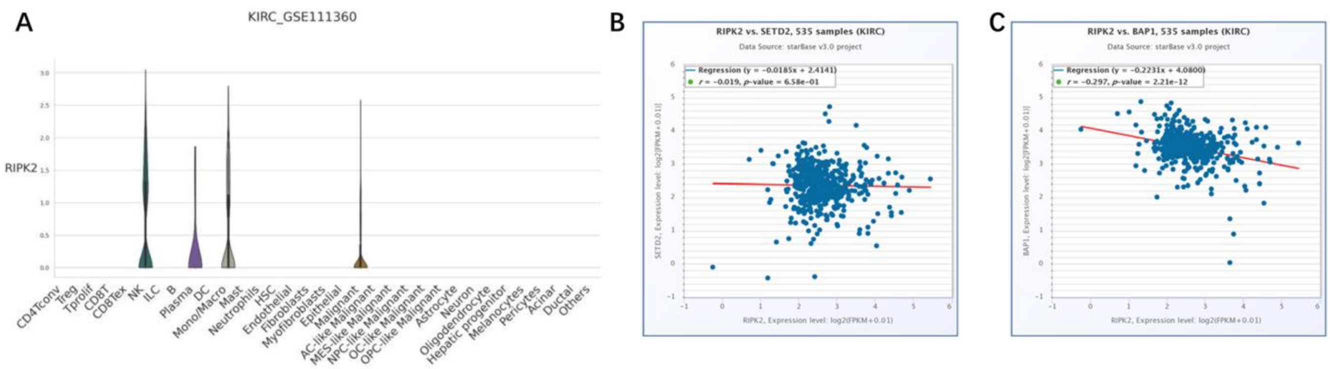

Supplementary Figure 5. Expression of RIPK2 in KIRC scRNA dataset and expression correlation between RIPK2 and SETD2 and BAP1. (**A**) Analysis of KIRC scRNA dataset (GSE111360) for the expression of RIPK2 among different groups of cells was shown in violin plot. (**B**) mRNA expression correlation between RIPK2 and SETD2 (r=-0.019) in TCGA KIRC patient dataset (http://starbase.sysu.edu.cn/). (**C**) mRNA expression correlation between RIPK2 and BAP1 (r=-0.297) in TCGA KIRC patient dataset (http://starbase.sysu.edu.cn/).
