# Supplementary material for: RIPK2 is an unfavorable prognosis marker and a potential therapeutic target in human kidney renal clear cell carcinoma
Source: Aging (Albany NY). 2021 Mar 31;13(7):10450–67. doi: 10.18632/aging.202808 (PMC8064209; doi:10.18632/aging.202808)
Supplement: Supplementary Table 1 [file aging-13-202808-s002.doc]

Supplementary Table 1. Differential expressed genes in *RIPK2* high expression samples when compared with low expression samples.

| **Differential expressed genes** | | |
| --- | --- | --- |
| Gene Symbol | log2FoldChange | log10Padj |
| KRT19 | 1.734136 | 7.262826 |
| ALDH3A1 | 1.946847 | 10.34631 |
| AKR1C1 | 2.375462 | 16.28553 |
| KRT8 | 1.586809 | 10.32265 |
| AKR1C2 | 1.805223 | 10.20635 |
| TFRC | 1.152887 | 16.21949 |
| NTRK2 | 2.446225 | 18.40578 |
| AKR1C3 | 2.246334 | 16.44457 |
| CES1 | 3.836025 | 24.55841 |
| NTS | 4.254118 | 18.44339 |
| ABCC1 | 1.093262 | 16.69049 |
| GPX2 | 1.388173 | 5.651226 |
| TXNRD1 | 1.675951 | 24.87209 |
| ABCC5 | 1.547662 | 24.57507 |
| G6PD | 1.197547 | 15.9818 |
| ALDH1A1 | 2.719221 | 21.03004 |
| SOX2 | 2.44381 | 27.15866 |
| GCLC | 1.306563 | 15.63759 |
| EPCAM | 2.051284 | 25.71868 |
| GSTM3 | 2.080577 | 17.38042 |
| EPHX1 | 1.121646 | 13.59325 |
| ALCAM | 1.169203 | 13.13734 |
| BPIFB1 | 1.923014 | 3.558532 |
| PGRMC1 | 1.125599 | 172.1202 |
| ADH7 | 1.92258 | 8.825702 |
| MUC5B | 1.24239 | 2.228216 |
| CYP4F11 | 2.567429 | 18.58002 |
| GPC3 | 3.302977 | 27.11896 |
| MYH11 | 2.018937 | 13.87136 |
| FADS2 | 1.331492 | 13.97123 |
| SLC7A11 | 1.63469 | 11.63713 |
| PTGR1 | 1.039547 | 9.035015 |
| GCLM | 1.500687 | 17.90685 |
| CYP2S1 | 1.060087 | 8.688053 |
| PHGDH | 1.32579 | 14.34629 |
| UPK1B | 1.32855 | 2.616171 |
| NUP210 | 1.084285 | 9.33439 |
| BMP7 | 1.154635 | 9.474918 |
| PRKX | 1.272682 | 20.70812 |
| ADAM23 | 1.587587 | 10.88559 |
| MAP1B | 1.274854 | 12.25959 |
| UCHL1 | 1.449311 | 6.44368 |
| MUC4 | 1.105918 | 2.834352 |
| KLHL24 | 1.064797 | 17.47893 |
| MGST1 | 1.285902 | 4.490454 |
| CYP4F3 | 2.968544 | 23.49706 |
| CALB1 | 1.788378 | 5.03234 |
| B4GALT4 | 1.044785 | 13.55427 |
| FZD7 | 1.612087 | 18.18212 |
| GSTM1 | 1.731794 | 4.567875 |
| ZBTB7C | 1.138538 | 7.112843 |
| FGFR2 | 1.105916 | 14.00685 |
| FADS1 | 1.117736 | 14.55704 |
| ABCA13 | 1.973037 | 16.17832 |
| WNK2 | 2.170385 | 16.97208 |
| C3orf58 | 1.269037 | 23.5392 |
| TRIM2 | 1.069746 | 11.03944 |
| GSTA1 | 3.664931 | 15.31641 |
| SDK1 | 1.21078 | 11.60023 |
| OLFM1 | 1.458529 | 9.165266 |
| TRIM16L | 1.506473 | 16.39619 |
| FGFBP2 | 3.611486 | 20.31595 |
| BCAT1 | 1.147449 | 8.530968 |
| SUSD4 | 1.220412 | 9.284424 |
| GABRP | 1.206989 | 3.00345 |
| NMRAL2P | 2.552285 | 20.17172 |
| PBX1 | 1.067204 | 8.061909 |
| CP | 1.351841 | 4.789409 |
| MEST | 1.099655 | 14.08571 |
| SOX13 | 1.042455 | 17.12509 |
| CBX2 | 1.212814 | 17.24874 |
| ICK | 1.049972 | 18.82703 |
| STC2 | 1.058371 | 9.395067 |
| EPS8 | 1.457826 | 18.33902 |
| TSPAN18 | 1.37368 | 12.44744 |
| C8orf4 | 1.548511 | 11.07849 |
| LRRC4 | 1.407537 | 7.524276 |
| GSTM4 | 1.423611 | 16.14327 |
| CCL20 | 1.009086 | 3.656928 |
| KRT7 | 1.064089 | 3.145975 |
| PIR | 1.024099 | 11.66492 |
| OSGIN1 | 1.244296 | 8.455955 |
| RAB6B | 2.431781 | 30.52757 |
| UGT1A7 | 3.895746 | 21.18574 |
| EDIL3 | 1.008154 | 6.400914 |
| RASSF9 | 1.984882 | 19.16277 |
| HEY1 | 1.612743 | 15.2933 |
| SAMD12 | 1.676525 | 12.49758 |
| CNTNAP2 | 1.404289 | 6.102244 |
| PODXL2 | 1.608955 | 11.7499 |
| NOS2 | 2.077692 | 11.91836 |
| PLEKHG4 | 1.12069 | 11.68406 |
| KCNS3 | 1.140055 | 12.85089 |
| CEL | 1.254036 | 3.977738 |
| RP1-27K12.2 | 4.980114 | 29.32268 |
| GLI2 | 1.801082 | 14.39955 |
| GSTM2 | 2.114979 | 19.81818 |
| KIAA1324 | 1.854872 | 12.40778 |
| SCN9A | 3.42795 | 34.21877 |
| ZDHHC2 | 1.569338 | 15.64765 |
| SLC4A11 | 1.060687 | 9.370929 |
| NRCAM | 1.1467 | 5.497206 |
| PRAME | 1.412292 | 3.676351 |
| SHISA2 | 1.388674 | 7.449363 |
| PANX2 | 1.971316 | 19.11125 |
| FREM2 | 2.715121 | 12.93022 |
| FOXA1 | 1.790411 | 10.53625 |
| KCNE3 | 2.472474 | 34.92486 |
| RAB3B | 2.032062 | 12.33413 |
| PTCH1 | 1.678403 | 28.28284 |
| PXYLP1 | 1.540998 | 23.14521 |
| MDGA1 | 1.18092 | 9.32476 |
| COL9A2 | 1.244838 | 7.756166 |
| TKTL1 | 3.315246 | 16.92573 |
| PTN | 1.212398 | 6.733219 |
| FRAS1 | 1.517488 | 12.06063 |
| ARHGEF26 | 2.305563 | 24.7669 |
| LAMA1 | 1.014121 | 4.261362 |
| LOXL4 | 1.584963 | 8.635442 |
| PCDH19 | 3.254268 | 30.41961 |
| PLAC8 | 1.54723 | 8.493798 |
| SEMA6A | 1.063089 | 6.961373 |
| SOX21 | 1.330794 | 6.152987 |
| CHPT1 | 1.214372 | 12.91316 |
| KLHL13 | 1.442005 | 27.14855 |
| TMEM116 | 2.108518 | 38.11952 |
| SLC35G1 | 2.153612 | 35.38097 |
| SOSTDC1 | 1.440722 | 4.94215 |
| MECOM | 1.116837 | 8.759576 |
| RP1-152L7.5 | 2.614263 | 27.2479 |
| TPTEP1 | 2.759428 | 20.35828 |
| NPR3 | 1.403016 | 8.194081 |
| SCUBE3 | 3.630422 | 43.29356 |
| MCF2L | 1.117649 | 11.78764 |
| LINC01206 | 3.483973 | 24.26289 |
| EPHA7 | 3.098789 | 20.91244 |
| OTX1 | 1.658024 | 21.44052 |
| IGF2BP3 | 1.004141 | 4.570616 |
| LDOC1 | 1.407047 | 11.04799 |
| NRN1 | 1.385519 | 9.370929 |
| CCDC190 | 1.962719 | 6.621114 |
| CYP26A1 | 3.811451 | 21.79846 |
| BEX4 | 1.291352 | 13.72865 |
| TMEM158 | 1.162015 | 11.11972 |
| BEX2 | 2.46959 | 30.10072 |
| UGT8 | 2.002141 | 10.45588 |
| TBX2 | 1.013948 | 8.663058 |
| WIF1 | 5.956046 | 42.57283 |
| GCNT2 | 2.065038 | 25.27578 |
| KIAA1549 | 1.706194 | 20.56561 |
| PNCK | 1.841233 | 11.28151 |
| MSMB | 1.447418 | 4.352633 |
| GDA | 1.650433 | 5.856178 |
| PARM1 | 1.123337 | 7.389499 |
| GPR160 | 1.87617 | 22.79184 |
| SDPR | 1.196197 | 7.654343 |
| ADD2 | 1.775826 | 9.030773 |
| MEX3A | 1.467473 | 17.35963 |
| CPLX2 | 5.050409 | 26.81099 |
| ARNT2 | 1.133081 | 6.048866 |
| IGF2BP1 | 1.363377 | 4.621224 |
| MRAP2 | 2.653339 | 25.7824 |
| SBK1 | 2.078064 | 20.01526 |
| SYCP2 | 2.344606 | 16.11822 |
| SIX4 | 1.292268 | 15.9678 |
| FAM83E | 1.070339 | 6.152588 |
| MIR9-3HG | 1.854804 | 12.59234 |
| PRSS21 | 1.244326 | 3.651047 |
| FGF19 | 4.572459 | 26.92924 |
| FLVCR1 | 1.044651 | 23.31469 |
| B4GALNT4 | 1.388242 | 8.301874 |
| SOX2-OT | 3.801594 | 52.73775 |
| MYO5C | 1.034047 | 7.065172 |
| ARFGEF3 | 1.94958 | 18.55662 |
| LGR6 | 1.888113 | 13.31784 |
| UGT1A6 | 2.732073 | 19.00803 |
| PELI2 | 1.471832 | 15.5405 |
| CHRM3 | 1.359841 | 5.374237 |
| FAM3B | 1.407678 | 4.786768 |
| CLIC6 | 1.674013 | 13.44391 |
| TSPYL5 | 1.49292 | 10.60576 |
| CHST7 | 1.199355 | 16.39274 |
| NEFH | 3.373892 | 34.84723 |
| ZBTB10 | 1.011785 | 8.937466 |
| KLHL23 | 1.577286 | 21.40761 |
| ALOX15 | 3.572293 | 30.22467 |
| MYB | 2.342558 | 26.65462 |
| SLC29A4 | 1.498005 | 13.02378 |
| ATP6V0E2 | 1.026188 | 8.630925 |
| SERPINI1 | 1.45977 | 13.58301 |
| AC005336.4 | 3.190639 | 20.49512 |
| C1orf115 | 1.027109 | 12.37967 |
| STAG3 | 1.871151 | 11.33017 |
| SEMA6D | 1.287084 | 8.12255 |
| MUC7 | -1.88144 | 2.679517 |
| CDSN | -2.38754 | 10.68538 |
| LMOD3 | -1.45083 | 3.762384 |
| ADAP2 | -1.23083 | 20.95644 |
| TCN1 | -1.07538 | 2.92453 |
| SRL | -1.46943 | 5.19485 |
| SPTLC3 | -1.32932 | 11.0731 |
| TRPV3 | -1.28066 | 9.343678 |
| LY6G6C | -1.0959 | 3.827151 |
| WFDC5 | -1.2517 | 6.380998 |
| PNLIPRP3 | -1.71239 | 6.145426 |
| CST6 | -1.46629 | 8.09435 |
| ANKRD22 | -1.01267 | 9.026141 |
| GNLY | -1.20765 | 7.601193 |
| LIPG | -1.28603 | 8.943794 |
| SPRR2C | -1.73381 | 7.750494 |
| BICDL2 | -1.00671 | 7.107925 |
| CWH43 | -1.38342 | 4.291382 |
| LYPD2 | -1.58878 | 5.137994 |
| ALOXE3 | -1.50951 | 10.36007 |
| USP2 | -1.87627 | 20.92203 |
| LCE3A | -2.6605 | 12.26533 |
| THSD1 | -1.09881 | 12.31412 |
| MYLPF | -1.4287 | 3.625583 |
| XIRP1 | -1.21079 | 3.881256 |
| CTC-518B2.8 | -1.47109 | 6.092933 |
| SH2D5 | -1.51883 | 10.30691 |
| NOD2 | -1.05311 | 9.524633 |
| CYP4F22 | -1.47751 | 5.911576 |
| VEGFC | -1.05201 | 6.396164 |
| DDN | -1.08669 | 4.08611 |
| SLC5A1 | -1.42763 | 7.065172 |
| MYOT | -1.14958 | 2.917728 |
| SERPINB7 | -1.17939 | 5.716391 |
| CD177 | -1.24266 | 3.838144 |
| KRT16P2 | -1.14819 | 3.485081 |
| LMOD2 | -1.6686 | 3.190774 |
| PGLYRP4 | -1.56316 | 11.37342 |
| CITED4 | -1.23381 | 9.297708 |
| C2orf54 | -1.29119 | 7.057402 |
| MROH6 | -1.12317 | 12.22338 |
| ATP1A2 | -1.28626 | 3.438595 |
| SLC28A3 | -1.31481 | 11.32987 |
| PRSS3 | -1.63591 | 7.194536 |
| CEACAM19 | -1.03959 | 9.052531 |
| PLA2G4D | -2.13971 | 11.01476 |
| IL18 | -1.01973 | 9.797688 |
| SPNS2 | -1.26095 | 10.55156 |
| BATF2 | -1.63477 | 12.07165 |
| MEF2C | -1.10473 | 6.423203 |
| FLG | -1.8705 | 9.146838 |
| PAQR5 | -1.35031 | 11.86735 |
| SCNN1D | -2.23249 | 21.24541 |
| EPGN | -1.68793 | 7.078565 |
| DEFB4A | -2.328 | 8.189624 |
| PRR9 | -1.8766 | 5.879613 |
| KLK14 | -1.35858 | 4.000439 |
| CYP26B1 | -1.1931 | 9.990277 |
| MUC21 | -1.48918 | 3.053351 |
| DEFB1 | -1.45884 | 7.681844 |
| SLC6A14 | -1.21182 | 4.368476 |
| ISG20 | -1.14343 | 12.70822 |
| TMEM184A | -1.03785 | 9.705877 |
| CASP1 | -1.03178 | 17.29445 |
| SPINK6 | -3.34478 | 18.85835 |
| TOR4A | -1.10702 | 11.33101 |
| GOLGA7B | -1.2585 | 8.731101 |
| GSDMA | -2.05745 | 14.19773 |
| GPR68 | -1.01355 | 10.32527 |
| SDR9C7 | -1.6645 | 8.829346 |
| ENO3 | -1.34461 | 6.282601 |
| FLRT3 | -1.31926 | 6.38017 |
| HSPB6 | -1.35811 | 5.704678 |
| TRDN | -1.49508 | 3.016529 |
| IFIT2 | -1.00608 | 6.280647 |
| TNNC2 | -1.60671 | 5.110283 |
| PYGM | -1.33013 | 4.466012 |
| WFDC12 | -2.70742 | 12.87134 |
| RIN1 | -1.00651 | 10.68064 |
| DDX60L | -1.07924 | 14.30603 |
| VSIG10L | -1.1085 | 5.845836 |
| ARHGEF37 | -1.04047 | 11.37761 |
| IL36G | -1.05642 | 4.45421 |
| MYOM3 | -1.77318 | 12.53629 |
| SLN | -1.87187 | 5.210609 |
| FAM25A | -2.19592 | 10.94738 |
| SDCBP2 | -1.0701 | 9.606454 |
| S100A12 | -1.89641 | 11.47285 |
| RNASE7 | -1.74651 | 11.84301 |
| ADAMTSL4 | -1.30549 | 13.10999 |
| CCL21 | -1.35992 | 6.969608 |
| XDH | -1.4348 | 14.1465 |
| ALS2CL | -1.21753 | 15.62574 |
| APOBEC3A | -1.4807 | 9.689054 |
| DUOXA1 | -1.13783 | 15.6765 |
| SDR16C5 | -1.18398 | 9.549552 |
| PTK6 | -1.16612 | 12.65084 |
| OASL | -1.0227 | 5.559069 |
| PRSS27 | -1.73272 | 11.83284 |
| MT1E | -1.03659 | 5.294628 |
| SPRR2F | -1.57029 | 5.332909 |
| TMEM40 | -1.31616 | 14.34172 |
| DHRS1 | -1.20477 | 19.40154 |
| STEAP4 | -1.46928 | 9.462901 |
| CDA | -1.5712 | 10.83586 |
| IL1B | -1.29337 | 9.017166 |
| EPSTI1 | -1.12025 | 10.68312 |
| STAC3 | -1.18648 | 4.667013 |
| EREG | -1.38964 | 5.229838 |
| SERPINB2 | -1.05415 | 4.57594 |
| DSC1 | -1.70629 | 5.623128 |
| LGALS7 | -1.91782 | 6.665786 |
| METRNL | -1.05306 | 14.73892 |
| KPRP | -3.00298 | 18.21483 |
| KLK12 | -1.78963 | 7.643195 |
| ALOX12B | -1.83391 | 9.502234 |
| ATP2A1 | -1.34268 | 4.874062 |
| CYSRT1 | -1.97356 | 15.63116 |
| LCE3E | -2.35651 | 10.81858 |
| MAL | -1.84186 | 7.023076 |
| EPS8L1 | -1.31769 | 12.37663 |
| HSPB7 | -1.40441 | 3.871829 |
| GSDMC | -1.23387 | 11.56645 |
| EEF1A2 | -1.38969 | 5.142687 |
| TNNC1 | -1.99323 | 6.469525 |
| SPINK7 | -3.53476 | 20.91244 |
| LGALSL | -1.27838 | 13.85287 |
| CASP4 | -1.04873 | 20.57436 |
| KCNK6 | -1.10824 | 16.61637 |
| IRF7 | -1.20638 | 16.04444 |
| GALNT6 | -1.02041 | 7.775433 |
| KRT78 | -1.36905 | 4.998428 |
| CLIC3 | -2.05159 | 21.42583 |
| CSRP3 | -1.73084 | 2.987012 |
| RRAD | -1.19871 | 5.769197 |
| IFIH1 | -1.01161 | 11.19888 |
| ASPRV1 | -1.5433 | 5.271111 |
| LGALS7B | -1.02448 | 3.553697 |
| TNNI2 | -1.51223 | 5.09306 |
| PTGS1 | -1.06547 | 7.325354 |
| KLHL41 | -1.6024 | 3.781765 |
| DDX58 | -1.23807 | 16.02192 |
| FAM46B | -1.41444 | 10.85889 |
| NELL2 | -1.03477 | 5.127319 |
| TMPRSS11D | -1.14306 | 5.285483 |
| C1orf116 | -1.00484 | 9.564325 |
| TMEM54 | -1.05689 | 14.4014 |
| KLK8 | -1.66338 | 9.99889 |
| C19orf33 | -1.53055 | 12.14933 |
| NIPAL4 | -1.23225 | 7.57752 |
| HOPX | -1.23015 | 7.24319 |
| SAA1 | -1.92579 | 13.23285 |
| CXCL11 | -2.30151 | 15.39743 |
| MYL2 | -1.52246 | 2.840923 |
| TRIM22 | -1.05545 | 11.05508 |
| TUBB2A | -1.44377 | 19.66349 |
| KRT16P6 | -1.83463 | 8.702665 |
| SCEL | -1.5753 | 9.988635 |
| MXD1 | -1.02662 | 12.36208 |
| TMEM79 | -1.14328 | 12.72236 |
| IFIT1 | -1.13134 | 7.223938 |
| RSAD2 | -1.47525 | 12.50449 |
| MYL1 | -1.67127 | 2.662797 |
| TCAP | -1.49109 | 4.185745 |
| MYBPC2 | -1.36183 | 3.128332 |
| LYPD5 | -2.09739 | 22.81162 |
| PLK2 | -1.0946 | 15.01514 |
| SERPINB4 | -1.61319 | 8.4521 |
| CARHSP1 | -1.02272 | 16.9834 |
| RHOD | -1.26463 | 12.69731 |
| TNNT3 | -1.14706 | 2.773802 |
| SLURP1 | -2.73302 | 14.77591 |
| SPRR2B | -2.31796 | 10.48087 |
| SULT2B1 | -1.65258 | 13.58734 |
| XIRP2 | -1.69956 | 2.761962 |
| CRCT1 | -2.41142 | 13.6772 |
| NRAP | -1.46316 | 2.38108 |
| CA2 | -1.22694 | 7.892191 |
| CASP14 | -1.76252 | 5.67602 |
| TNNI1 | -1.49033 | 5.27444 |
| UPP1 | -1.34821 | 12.45906 |
| DUOX1 | -1.20094 | 17.44643 |
| PLA2G4E | -1.84428 | 11.60809 |
| EPPK1 | -1.3795 | 14.16513 |
| NCCRP1 | -1.47421 | 8.62662 |
| KLK13 | -1.58003 | 6.822492 |
| IFIT3 | -1.23772 | 9.673958 |
| ACTN2 | -1.36092 | 2.879429 |
| KRT80 | -1.25541 | 9.237582 |
| IL36RN | -1.70904 | 10.29641 |
| PDZK1IP1 | -1.50518 | 11.05632 |
| FLNC | -1.20208 | 4.774351 |
| MMP10 | -1.24079 | 4.511385 |
| MB | -1.36171 | 3.779908 |
| HEPHL1 | -1.42746 | 5.914009 |
| ECM1 | -1.06569 | 7.850345 |
| TNNT1 | -1.81064 | 12.00991 |
| C10orf99 | -1.74841 | 11.58603 |
| KLK6 | -1.75409 | 8.495547 |
| KRT75 | -2.03215 | 11.25338 |
| GBP5 | -1.6778 | 11.78552 |
| GBP1 | -1.02288 | 7.543392 |
| KLK5 | -1.66544 | 6.951198 |
| MYH7 | -1.76481 | 4.015962 |
| LCN2 | -1.11521 | 5.500862 |
| LCE3D | -2.38242 | 11.36646 |
| SERPINB3 | -1.38467 | 7.103623 |
| CXCL10 | -1.46981 | 6.98604 |
| SAMD9 | -1.14686 | 11.31328 |
| IGFBP6 | -1.33321 | 8.005854 |
| CLTB | -1.20117 | 17.04198 |
| CRABP2 | -1.20588 | 9.287088 |
| S100A7A | -1.71009 | 5.720971 |
| KLK7 | -1.88856 | 10.51218 |
| SERPINB1 | -1.05317 | 11.50162 |
| CRNN | -2.8179 | 11.74186 |
| NEB | -1.31067 | 3.992377 |
| CKM | -1.16552 | 2.435753 |
| SPINK5 | -1.40798 | 7.278194 |
| A2ML1 | -1.21527 | 5.772497 |
| PKP3 | -1.11359 | 17.4924 |
| SPRR2G | -2.16761 | 9.216399 |
| LYNX1 | -2.48684 | 25.15685 |
| GJB6 | -1.21024 | 6.513012 |
| TUBA4A | -1.25497 | 15.5579 |
| IFI6 | -1.11989 | 7.661942 |
| ISG15 | -1.60549 | 12.02464 |
| TYMP | -1.10203 | 13.11397 |
| CALML3 | -1.15009 | 8.687624 |
| TGM3 | -2.55732 | 11.1246 |
| IL1RN | -1.49388 | 14.46823 |
| S100A14 | -1.03309 | 8.365933 |
| FABP4 | -3.09529 | 17.44216 |
| KLK10 | -1.37843 | 7.458248 |
| FAM83A | -1.59289 | 13.03239 |
| MT2A | -1.30882 | 9.734451 |
| SPRR3 | -1.07531 | 2.599216 |
| CNFN | -2.31384 | 17.03746 |
| MMP1 | -1.05605 | 4.703382 |
| LYPD3 | -1.06359 | 8.626478 |
| IFI27 | -1.01335 | 8.446658 |
| KRT10 | -1.00069 | 2.967278 |
| MYH2 | -1.58019 | 2.45821 |
| IVL | -1.7385 | 11.52978 |
| ACTA1 | -1.2 | 2.281839 |
| TGM1 | -1.90994 | 13.9834 |
| DMKN | -1.73189 | 12.51952 |
| LY6D | -1.20593 | 5.471921 |
| RHCG | -1.75779 | 9.228408 |
| DES | -1.16143 | 2.131687 |
| TMSB10 | -1.01702 | 10.40222 |
| KRTDAP | -1.95918 | 8.998131 |
| SPRR2D | -1.92068 | 12.54149 |
| KRT1 | -1.09431 | 2.120549 |
| CSTA | -1.43649 | 11.71907 |
| DSG1 | -2.0799 | 9.93613 |
| FABP5 | -1.35603 | 9.796946 |
| SLPI | -1.76106 | 13.7998 |
| DSC2 | -1.34399 | 13.53859 |
| SPRR1A | -1.69329 | 8.43289 |
| WARS | -1.39952 | 13.05892 |
| SPRR2E | -2.20216 | 12.33455 |
| SPRR2A | -2.06813 | 13.13248 |
| CSTB | -1.24875 | 12.72358 |
| COL17A1 | -1.01513 | 5.898872 |
| S100A7 | -2.04466 | 10.52936 |
| AQP3 | -1.53503 | 10.94471 |
| SBSN | -1.83615 | 9.227774 |
| GJB2 | -1.59086 | 13.35252 |
| S100A2 | -1.24761 | 10.76647 |
| SPRR1B | -1.87879 | 13.27643 |
| KRT13 | -1.58413 | 5.329457 |
| ANXA1 | -1.34538 | 16.94481 |
| PI3 | -1.79669 | 7.206825 |
| S100A8 | -1.79102 | 10.66702 |
| SFN | -1.41497 | 15.62471 |
| KRT6C | -2.20139 | 11.74186 |
| S100A9 | -1.7469 | 13.49656 |
| KRT6B | -1.95253 | 15.94865 |
| KRT17 | -1.24534 | 10.61439 |
| KRT6A | -1.0555 | 8.418953 |
| KRT16 | -1.90084 | 9.468889 |
| KRT14 | -1.7052 | 13.47898 |
